# Incidence of Induction Toxicities in Childhood Acute Lymphoblastic Leukaemia in Ghana

**DOI:** 10.1155/ah/5787036

**Published:** 2026-06-23

**Authors:** Lily Gloria Tagoe, Emmanuella Amoako, Ernestina Schandorf, Lorna A. Renner, Catherine Segbefia, Kokou Amegan-Aho, Jennifer Welbeck, Yvonne Dei-Adomakoh

**Affiliations:** ^1^ Department of Child Health, Korle Bu Teaching Hospital, Accra, Ghana, kbth.gov.gh; ^2^ Department of Child Health, University of Ghana Medical School, Accra, Ghana, ug.edu.gh; ^3^ Department of Haematology, University of Ghana Medical School, Accra, Ghana, ug.edu.gh

**Keywords:** acute lymphoblastic leukaemia, children, induction, low-middle-income country, supportive care, treatment toxicities

## Abstract

**Introduction:**

Due to limited supportive care, low‐ and middle‐income countries balance delivering intensive, curative chemotherapy with the risk of treatment‐related toxicity. Acute lymphoblastic leukaemia (ALL) is the commonest childhood cancer, and induction is the highest risk period for morbidity and mortality. The study describes induction toxicities among children with ALL at Korle Bu Teaching Hospital, Ghana.

**Methods:**

This hospital‐based cohort study involved children aged 1–17 years newly diagnosed with ALL from August 2021 to January 2023. Demographic, clinical and laboratory data were recorded. Descriptive statistics (median [interquartile range]) were used to summarise continuous variables. Frequency and percentages were used to describe categorical variables. Toxicities were graded using the Common Terminology Criteria for Adverse Events, Version 5.0.

**Results:**

Thirty‐one children were included. Median age was 5 years (interquartile range [IQR]: 3–10 years). All 31 had at least one induction toxicity of any grade, and 29 (93.4%) had at least one severe (Grade ≥ 3). The most common toxicities were anaemia, thrombocytopenia, sepsis, febrile neutropenia, mucositis and hypertension; bleeding, acute pancreatitis, seizures and hepatotoxicity were less common. Induction mortality rate was 19.3%. There was no association found between age, sex, initial white cell count, treatment protocol and development of any severe toxicity.

**Conclusion:**

Severe induction toxicities were common in our cohort with unacceptably high mortality, underscoring the need to prioritise enhanced bedside clinical monitoring for hypertension, improved access to blood products and optimal infection prevention and control practices. Further research exploring the relationship between induction toxicities and patient outcomes is essential.

## 1. Introduction

Chemotherapy‐related toxicity is a major cause of mortality and both short and long‐term morbidity in patients treated for ALL [1]. The overall risk of a child with ALL developing at least one serious adverse event with first‐line therapy is 30%–50% [2].

Toxicities can occur throughout the course of treatment but more often during induction [3, 4] from the disease burden and the treatment intensity required to induce remission.

Toxicities which occur during ALL treatment include infections, febrile neutropenia, mucositis, pancreatitis, steroid‐induced hyperglycaemia, asparaginase hypersensitivity, tumour lysis syndrome (TLS), neurotoxicity, thrombosis and death [5].

Toxicities such as infections, febrile neutropenia and mucositis occur from bone marrow suppression caused by chemotherapy or the disease itself. Some toxicities are directly attributed to specific chemotherapy. For example, the development of acute pancreatitis during ALL treatment is related to the use of asparaginase, occurring in about 2%–18% of cases [6].

Some toxicities result in short‐ and long‐term morbidity, while others can be fatal [1]. Mortality during induction for childhood ALL accounts for about half of all treatment‐related deaths. This may be due to prolonged episodes of bone marrow suppression, either from the effects of chemotherapy or from disease [7].

Factors which have been evaluated in association with increased risk of treatment‐related toxicities and mortality include age, sex and white cell count. For example, in the UKALL 2003 study, older age (≥ 10 years) was significantly associated with increased risk of serious treatment‐related toxicities [5].

Treatment‐related toxicities can lead to protocol deviations including treatment delays, interruptions and dose reductions. These can compromise outcomes and increase the risk of relapse and treatment failure [2, 3].

In low‐ and middle‐income countries (LMICs) where supportive care is limited, there is a need to balance the delivery of intensive, curative chemotherapy with the risk of treatment‐related morbidity and mortality.

The aim of the study was to determine the incidence and severity of induction toxicities and factors associated with severe (≥ Grade 3) toxicities in children with ALL managed at the paediatric oncology unit (POU) of Korle Bu Teaching Hospital (KBTH), Accra, Ghana.

## 2. Methods

This was a hospital‐based cohort study involving children aged 1–17 years, newly diagnosed with ALL, receiving care at the POU, KBTH, between August 2021 and January 2023. KBTH, the largest national referral teaching hospital, houses the POU, comprising a 22‐bed inpatient ward and a 12‐bed outpatient day care. The POU serves as a primary referral centre for paediatric oncology cases under 18 years of age, both nationally and from neighbouring West African countries, including Togo, Liberia and Sierra Leone. The annual number of newly diagnosed patients in the unit is 200, with an average of 26 new ALL diagnoses per year.

Diagnostic confirmation of ALL was established in all participants using baseline bone marrow aspirate (BMA) or peripheral blood film evaluation. Immunophenotypic characterisation by flow cytometry was offered; however, due to financial constraints associated with external laboratory processing, this analysis was performed selectively.

Treatment protocols varied during the study period. Prior to January 2022, a modified UKALL 97/99 protocol was used. Subsequently, the unit transitioned to the locally developed Ghana_ALL protocol. In addition to the National Cancer Institute (NCI) risk stratification, the Ghana_ALL protocol incorporates additional high‐risk criteria, including steroid pretreatment, indeterminate central nervous system (CNS) status at Day 1 and testicular or CNS involvement. Notably, the protocol features a modification of anthracycline administration, commencing from Day 8, in contrast to the weekly administration from Day 1 utilized in the UKALL protocol (Table 1).

**TABLE 1:** Induction protocol (Ghana_ALL).

B‐ALL		T‐ALL
SR	HR	
Vincristine 1.5 mg/m^2^/dose (max. 2 mg) IV Days 1, 8, 15, 2 and 29		
Doxo/Daunorubicin 25 mg/m^2^/dose IV Day 8	Doxo/Daunorubicin 25 mg/m^2^/dose IV Days 8, 22 and 29	
Prednisolone 40 mg/m^2^/day PO in 2 divided doses Days 1–28	Prednisolone 60 mg/m^2^/day PO in 2 divided doses Days 1–28	Dexamethasone 10 mg/m^2^/day PO in 2 divided doses Days 1–7 and Days 15–21
Triple Intrathecal (age‐based dosing) on Days 1 and 29		
PEG‐Asparaginase 2500 units/m^2^/dose IM Day 4 (max. 3750 units)		

Supportive care for patients in the study was prescribed by the attending team, according to the KBTH POU Supportive Care guidelines (2021). Blood product transfusions were prescribed according to the unit guidelines, which generally aligns with a restrictive strategy (haemoglobin trigger of < 7.0 g/dL for red blood cells and a platelet count of < 10 × 10^6^/L for prophylactic transfusions). None of the children in the study received antimicrobial prophylaxis, as per standard unit practice for children with ALL. All febrile or ill patients on chemotherapy were treated with a presumed diagnosis of febrile neutropenia at presentation. Samples were taken for blood culture, and they were started on empiric intravenous antibiotics, ideally within an hour of presentation.

The study excluded children with ALL who had received prior treatment including steroids, who were offered upfront palliative care or whose caregivers refused treatment. Ethical approval was received from the KBTH Institutional Review Board (KBTH‐IRB/00075/2021), and the caregivers of all study participants gave written consent.

All enrolled participants were followed up for the duration of induction (29 days for standard risk and 36 days for high risk). During the first week of induction, data were collected daily from Day 1 of induction to Day 3, during which patients were on admission at the POU. After discharge, participants were followed up at their scheduled weekly outpatient visits during which chemotherapy was administered as per the treatment protocol. Participants who reported at the outpatient Day Care Unit for unscheduled visits on account of ill health also had data collected on those days.

At each follow‐up visit (scheduled or unscheduled), participants had data collected on symptoms and signs of toxicities. Symptoms of toxicities elicited included fever, vomiting, constipation, oral ulcers, abdominal pain, diarrhoea, bleeding, rash, excessive urination, thirst, headache, numbness, seizures, abnormal gait and difficulty in breathing. The participants were examined for signs of toxicities. Examination findings included vital signs (heart rate, blood pressure, respiratory rate and oxygen saturation), pallor, jaundice, examination of the oral mucosa for erythema and/or ulcers, abdominal examination for tenderness and masses, and a motor neurological exam. Data collected at each visit were reflective of symptoms since the time of last assessment.

The timing of routine laboratory investigations is shown in Table 2. Blood and urine cultures and serum amylase were done when clinically indicated.

**TABLE 2:** Schedule for follow‐up of laboratory evaluations.

Investigation	Days							
	Baseline	During chemo (days)						
		2	3	8	15	22	29	36
			±2	±4	±4	±4	±4	±4
FBC	X			X	X	X	X	X
Peripheral blood film	X			X				
BUE, Cr, Ca, PO_4_	X		X					
Uric acid	X		X					
LDH	X							
LFT	X							
RBS	X	X	X	X	X	X	X	X
BMA (morphology)	X						X	X

*Note:* BUE, Cr—blood urea and creatinine; Ca—serum calcium; PO4—serum phosphate; LDH—lactate dehydrogenase.

Abbreviations: FBC, full blood count; LFT, liver function test; RBS, random blood sugar.

End of induction BMA was done on day 29 (for standard risk) and day 36 (for high risk) to assess bone marrow recovery and morphologic remission status.

All toxicities were graded according to the NCI Common Terminology Criteria for Adverse Events (CTCAE) v5.0 classification [8]. For participants who experienced more than one episode of a particular toxicity, only the highest grade encountered was recorded. Toxicity event was defined as either the first episode or the highest grade of the toxicity, whichever occurred later.

Demographic, clinical and laboratory data of the participants were recorded and imported into IBM SPSS Version 25 for analysis. Descriptive statistics (median [interquartile range]) were used to summarise continuous variables. Frequency and percentages were used to describe categorical variables. Analysis. The nonparametric Mann–Whitney *U* test was used to test the association between the factors which were continuous variables (age and white cell count) and the development of severe toxicities. The Fischer exact test and Pearson chi‐square test were used to determine the association between factors which were categorical variables (age category, sex and type of treatment protocol) and the development of severe toxicities. All statistical analyses were considered significant at an alpha value of 0.05.

Treatment‐related toxicities assessed for during the period of induction were hypertension, hyperglycaemia, pancreatitis, GI toxicities—mucositis, nausea and vomiting, diarrhoea, constipation, CNS neurotoxicity—seizures, strokes, bleeding, febrile neutropenia, sepsis, TLS and anaemia. The definitions used for the study are shown in Supporting Table S1.

## 3. Results

A total of 55 children were assessed for study eligibility between August 2021 and January 2023. Of these, 21 were excluded due to reasons such as other diagnoses (*n* = 14), refusal of treatment (*n* = 2), with 34 being recruited. A further 3 patients were excluded after review of diagnosis, with 31 being finally included in the study.

Participant ages ranged from 1 to 13 years, with a median of 5 years (IQR: 3–10). Older participants (≥ 10 years) comprised 29% of the cohort, and males constituted 58.1%. Baseline white blood cell (WBC) counts varied widely, from 1.90 to 625.78 × 10^9/L, with a median of 98.37 × 10^9^/L (IQR: 11.89–232.0 × 10^9^/L). Over half (58%) of participants presented with a WBC ≥ 50 × 10^9^/L. The majority of the participants (74%) were treated with the locally developed Ghana_ALL protocol (SR–17%; HR–83%).

All participants (100%) experienced at least one toxicity during the period of induction, with an average of 5.3 ± 2.0 toxicity events per participant. Out of the 31 participants, 29 (93.4%) developed at least one severe toxicity, with an average of 3.7 ± 2.0 severe toxicity events per participant. The above values for toxicity events are presented as mean ± standard deviation. The most common toxicity was anaemia (Table 3). Anaemia was also the toxicity with the highest proportion of severe episodes, occurring in 74% of the study cohort. The highest nonhaematological toxicity was mucositis (12 participants; 38%). All 12 participants with mucositis, however, had Grade 2 or less toxicity.

**TABLE 3:** Frequency and grade of toxicities developed during induction.

Toxicity events^∗^	CTCAE grade					All toxicities	Severe toxicities (≥ Grade 3)
	1	2	3	4	5	Number (%)	Number (%)
Anaemia	0	4	18	3	2	27 (87.1)	23 (74.2)
Thrombocytopenia	2	2	2	19	0	25 (80.6)	21 (67.7)
Sepsis	0	0	15	2	3	20 (64.5)	20 (64.5)
Febrile neutropenia	0	0	15	4	0	19 (61.3)	19 (61.3)
Vomiting	2	4	5	1	0	12 (38.7)	6 (19.3)
Mucositis	4	8	0	0	0	12 (38.7)	0
Hypertension	0	5	5	1	0	11 (35.5)	6 (19.3)
Gastritis	1	6	3	0	0	10 (32.3)	3 (9.7)
Diarrhoea	6	2	0	1	0	9 (29.0)	1 (3.2)
Hyperglycaemia	0	0	4	0	0	4 (12.9)	4 (12.9)
Tumour lysis syndrome	0	0	4	0	0	4 (12.9)	4 (12.9)
Constipation	1	1	2	0	0	4 (12.9)	2 (6.4)
Stroke	0	0	2	1	0	3 (9.7)	3 (9.7)
Seizures	0	0	1	1	0	2 (6.5)	2 (6.5)
Hepatotoxicity	0	2	0	0	0	2 (6.5)	0
Bleeding	0	0	0	0	1	1 (3.2)	1 (3.2)
Pancreatitis	0	0	1	0	0	1 (3.2)	1 (3.2)
TOTAL	18	35	65	32	6^∗∗^	153	102

^∗^A single participant may have developed more than one toxicity (average is 5.3 toxicity events per participant).

^∗∗^One participant developed 2 different Grade 5 toxicities; one participant died from a nontreatment related cause; hence total deaths were 6.

**FIGURE 1:**
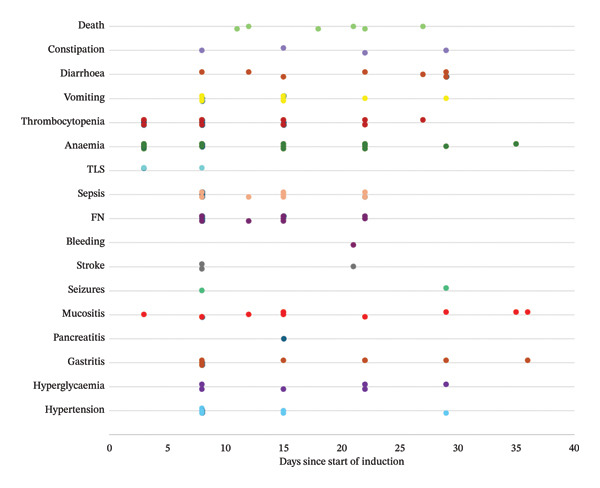
Toxicities in relation to days since start of induction treatment.

The majority of the toxicities occurred during the first 2 weeks of induction. Figure 1 shows the toxicities in relation to time of induction.

Of the 11 children who developed hypertension, 1 had Grade 4 toxicity and presented with severe hypertension and seizures. Brain imaging (CT scan) was normal, and a cause was not found, so he was subsequently treated as CNS positive empirically. Median time to development of hypertension was 8 days.

All 4 participants (13%) who developed hyperglycaemia required insulin which was stopped prior to the end of induction.

The participants who developed TLS were NCI high risk, with 3 out of 4 presenting with hyperleukocytosis. All cases had laboratory TLS, and 3 had LDH values above 1000 IU/L.

There was a high incidence of neurological toxicities (16.2%) in the study—3 participants developed stroke. In 1, this occurred shortly prior to death. This was attributed to an acute‐onset intracranial bleed. The other 2 participants eventually experienced full recovery and had no residual focal neurological deficit. Two (2) participants had seizures—one had brain imaging done (CT scan) which showed no abnormalities.

**FIGURE 2:**
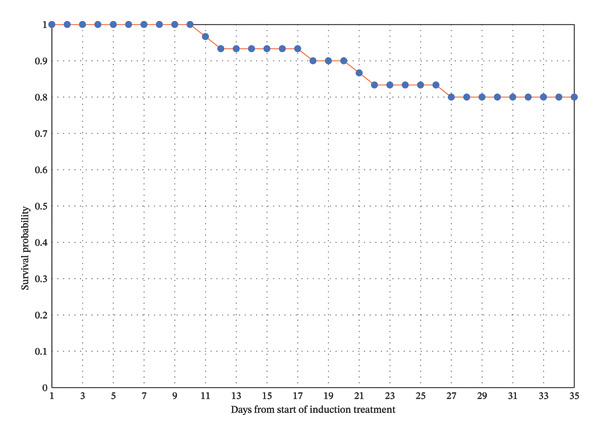
Time to Induction death.

The induction mortality rate was 19.3% (6 deaths). Three (9.7%) were due to infection. One of these infections was further complicated by severe anaemia. One child died from a suspected intracranial bleed linked to severe thrombocytopenia, and another from severe anaemia. One death, during the first 2 weeks of treatment, was unrelated to treatment, caused by aspiration pneumonitis. Of the 6 total deaths, 2 (33%) occurred during early induction (first 2 weeks), and the others occurred in late induction (Figure 2). All deaths occurred in hospital.

Almost all participants (93.5%) developed at least one severe toxicity. Therefore, the selected factors were correlated with the cumulative incidence of individual toxicities rather than the presence or absence of severe toxicity in general. The development of severe hyperglycaemia was significantly associated with age (*p* = 0.039) on Mann–Whitney U analysis. There was, however, no association found between the age categories (1–10 years and ≥ 10 years) and the development of severe hyperglycaemia. There was no association found between age and development of any other severe toxicities.

There was also no significant association found between initial white cell count, type of treatment protocol, sex and the development of any of the severe toxicities during the period of induction.

## 4. Discussion

During induction, all participants experienced at least one treatment‐related toxicity. The universal occurrence observed in this cohort, despite the utilisation of a modified regimen, underscores the critical importance of prioritising enhanced supportive care, regardless of perceived treatment intensity.

The highest toxicities encountered were haematological toxicities and infections. This is expected given the inherent mechanisms of action of chemotherapy and the underlying pathophysiology of leukaemia. Chemotherapy‐induced neutropenia is also a major risk factor for the development of infection. In a Brazilian paediatric cohort, 19.3% of patients had a laboratory‐documented bloodstream infection, with an additional 3.9% having clinical sepsis without a blood isolate during induction [9]. In contrast, the incidence of sepsis in this study cohort was much higher, reflecting the need for enhanced infection prevention and control practices. Additionally, other factors may have been implicated in the increased rates. Poor nutritional status is known to be independently associated with increased risk of treatment‐related toxicity, especially myelosuppression and infections, and poorer overall survival [10, 11]. The influence of nutritional status was not explored in this study. However, a high baseline prevalence of wasting at diagnosis (40.5%) was found using arm anthropometry in a previous study conducted in the same unit by Salifu et al. [12].

Of the participants, 12.9% developed TLS during induction, all of whom had laboratory TLS. All received conservative management and none required renal replacement therapy. In contrast, a retrospective study among children in Turkey, less than 1% developed TLS [13]. The difference may be attributed to the burden of disease at presentation as over half (58.1%) of participants had an initial WBC ≥ 50 × 10^9^/L compared to only 13% in the Turkish study. Conversely, a much higher incidence of TLS (62.6%) was found in Pakistan [14] and Egypt [13], where 38.5% and 45% of patients, respectively, had baseline WBC ≥ 50 × 10^9^/L [15]. The POU in KBTH employs strategies to reduce TLS in at‐risk patients, such as patients presenting with hyperleukocytosis. This includes hyperhydration of at least 3 L/m^2^/day, allopurinol and prephase steroids with gradually increasing doses over up to a week, prior to the start of formal induction chemotherapy. Considering the proportion of participants with high white cell counts at presentation, the relatively low incidence of TLS in this study cohort supports the effectiveness of our TLS prophylactic strategies. This model could be adapted by other LMICs with a high burden of high‐risk ALL.

Hypertension occurred in 35.4% of participants in the study, all requiring oral antihypertensive treatment, a rate lower than reported in a United States study (45%) but slightly higher than in India (29%) [16, 17]. The severity of hypertension was not graded in either of the comparator studies [16, 17], but in the Indian study [17], one patient had PRES, implying a severe grade of hypertension. In the current cohort, even though one patient with hypertension presented with seizures, an underlying cause was not found, probably because an MRI or CT with contrast‐enhanced venography was not done to differentiate between PRES, CSVT and other alternatives. The ability to perform MRI scans is often limited by cost or the need for sedation which may preclude timely imaging in emergency situations. The high incidence of hypertension during induction, however, emphasises the need for blood pressure monitoring during induction.

Hyperglycaemia occurred in 4 participants (12.9%), 3 of whom were adolescents. This was in keeping with rates found among Hispanic children in the United States (11.0%) [18]. A much higher prevalence of hyperglycaemia (35%) was found in another American retrospective study [19]. In the present study cohort, all affected patients received insulin. In contrast, in the former American cohort, less than half (47%) of the children were treated with insulin [18]. The difference in the rate of insulin use may have been due to the lack of clear‐cut criteria for starting insulin in patients with ALL whose hyperglycaemia is generally transient. In the KBTH POU, patients are started on insulin once hyperglycaemia (≥ 11.1 mmol/L) is present on two or more occasions, irrespective of the presence or severity of associated symptoms. Drugs used in induction such as steroids and asparaginase uniformly cause hyperglycaemia. The requirement for insulin in this cohort prolongs hospital stays due to the requisite close glycaemic monitoring for dose titration and patient education on insulin administration. This extended duration has considerable financial and logistical consequences, especially within the limited capacity of the POU. This current strategy may require a review to limit insulin use to patients with severe hyperglycaemia, as is the practice in other settings [20].

Within this study cohort, a limited number of participants (*n* = 4, 12.9%) experienced constipation, with half of these cases classified as severe. The observed incidence of constipation is notably lower than the estimated prevalence of 16% among patients with cancer [21]. Both vincristine and morphine—a commonly used analgesic—can cause constipation. Differentiating between these would require consideration of which patients are on concurrent opioids and adequacy of laxative dosing.

The incidence of neurological toxicity in this study was notably high at 16.1% (all ≥ Grade 3), exceeding rates reported in literature (4%–11% in Poland, 3.9% in Turkey and 2.3% in Argentina) [22–24]. Even though the latter 2 studies reported on seizures and focal motor deficits, their retrospective nature may have led to the relatively lower prevalence found. The criteria used to diagnose neurotoxicity may also have influenced the findings. For example, the Turkish study excluded patients with leukemic infiltration [23].

Stroke occurred in 3 participants (9.7%) in this study cohort. Stroke‐like symptoms have also been described in relation to the administration of methotrexate through both intravenous and intrathecal routes [22, 25]. In the United States, out of 369 children with ALL, 6 (1.6%) developed stroke‐like symptoms, which were attributed to methotrexate [25]. This study, however, was not limited to induction. As intrathecal methotrexate is given routinely during induction, it may have also contributed to neurotoxicity in the present study cohort.

Seizures occurred in 2 participants (6.4%) with no clear aetiology, similar to a Nordic study (5.6%) [26], where PRES and sinus venous thrombosis accounted for the majority of the seizures (63.4%), but 20% remained unexplained. The lack of further imaging (MRI and CT venogram) in our cohort may have limited the identification of underlying causes such as PRES and sinus venous thrombosis, respectively. Establishing a definite cause for the seizures would have enabled effective directed therapy, especially in the case of the latter, leading to improved outcomes. Almost all mortalities observed were directly attributable to infection‐related or haematologic toxicities, rather than progression of the underlying malignancy. The clustering of these deaths during late induction strongly suggests a treatment‐related aetiology, highlighting the acute vulnerability of patients during the most intensive phase of chemotherapy. The high incidence of toxic death, particularly from infection and haematologic complications, is a common theme across many LMICs. In Pakistan, a similar induction mortality rate (19.2%) was found, with the majority of deaths being due to infection‐related causes [27]. This has implications for the achievement of the World Health Organization’s Global Initiative for Childhood Cancer goal of 60% survival by 2030. While the POU at KBTH possesses foundational guidelines for critical supportive care issues—specifically the management of febrile neutropenia, and blood and blood product support for severe anaemia, and thrombocytopenia—it is important to explore how systemic delays in care ultimately translate these toxicities into preventable deaths. More frequent checks of blood counts during late induction may enable earlier detection of profound neutropenia. However, this approach will increase the financial burden on families (e.g., transportation costs and lab fees.) with a limited effect on patient outcomes if underlying gaps in supportive care, such as access to antibiotics and adequate clinical monitoring, are not addressed.

In this study, time to administration of antibiotics was not determined. However, this may have had an impact on infection outcomes, as found in a study in Bangladesh, where adverse events, including in‐hospital mortality, were associated with prolonged time to antibiotic administration (> 60 min) in children with febrile neutropenia [28]. The impact of antimicrobial prophylaxis in decreasing toxic deaths, though not currently standard practice in the KBTH POU, has also been explored in other studies, with varying conclusions. Contrary to findings of a meta‐analysis showing decreased mortality with fluoroquinolone‐based prophylaxis in patients with neutropenia, a randomised trial in Indonesia showed higher death rates [29, 30].

There was no statistically significant association between age and any toxicity which may be partly due to the age limit of the POU with disproportionately fewer adolescents seen at the study site. Sex was also not a predictor of development of severe toxicity in this study cohort. Although all participants who developed TLS in the present study had a high white cell count (≥ 91 × 10^9^/L), white cell count was not significantly associated with the development of TLS (≥ Grade 3). In contrast, many studies have reported a positive association between elevated white cell count and TLS [13, 15, 31]. Failure to establish an association in the present study cohort is likely because over half of participants had WBC > 50 × 10^9^/L at presentation.

The small sample size limited our power to detect statistically significant associations between the factors explored and severe toxicities and may restrict the generalisability of our findings. Future work should prioritise multicentre collaboration to achieve a larger sample size.

## 5. Conclusion

In Ghanaian children with ALL, induction therapy is associated with a substantial burden of toxicity, primarily anaemia, thrombocytopenia, sepsis, febrile neutropenia, mucositis and hypertension. While less frequent, bleeding, acute pancreatitis, seizures and hepatotoxicity also occurred. Nearly all patients experienced at least one severe (Grade 3 or higher) toxicity during induction, as classified by the CTCAE. Despite the small sample size, which may have limited the ability to detect associations between patient characteristics and severe toxicity, the findings are representative of the unit’s patient population and highlight the critical need for enhanced monitoring, improved supportive care to mitigate morbidity and mortality and optimised care within the constraints of limited inpatient capacity. Future research correlating induction toxicities with patient outcomes is warranted.

## Author Contributions

Catherine Segbefia had full access to all of the data in this study and takes complete responsibility for the integrity of the data and the accuracy of the data analysis.

## Funding

The funding for the study was provided by World Child Cancer as part of a paediatric fellowship educational grant.

## Disclosure

The manuscript was presented at the 55^th^ Congress of the International Society of Paediatric Oncology. All authors have read and approved the final version of the manuscript.

## Conflicts of Interest

The authors declare no conflicts of interest.

## Supporting Information

Additional supporting information can be found online in the Supporting Information section.

## Supporting information


**Supporting Information** Supporting Table 1 (S1) provides the definitions of toxicities used in this study.

## Data Availability

The data that support the findings of this study are available from the corresponding author upon reasonable request.
