# Supplementary material for: Incidence of Induction Toxicities in Childhood Acute Lymphoblastic Leukaemia in Ghana
Source: Adv Hematol. 2026 Jun 23;2026:5787036. doi: 10.1155/ah/5787036 (PMC13288359; doi:10.1155/ah/5787036)
Supplement: Supplementary file 1 — Supporting Information Supporting Table 1 (S1) provides the definitions of toxicities used in this study. [file AH-2026-5787036-s001.docx]

SUPPORTING INFORMATION MATERIAL

SUPPLEMENTAL TABLE S1 Definitions of toxicities

| Toxicity | Definition |
| --- | --- |
| Hypertension | Blood pressure greater than the 95th centile for sex, age and height for children and ≥130/80mmHg for adolescents ≥13 years, measured on at least 3 occasions.^32^  Blood pressure was measured for all participants using the Omron® digital sphygmomanometer Model M7 Intelli iT (HEM-7322T-E) with appropriate-sized paediatric cuff. |
| Hyperglycaemia | Random blood sugar level of ≥ 11.1mmol/l on a capillary finger-prick sample on at least 2 separate occasions with or without symptoms.^33^ |
| Anaemia | Either of the following, using a combination of the World Health Organisation (WHO), National Comprehensive Cancer Network (NCCN) and NCI CTCAE definitions^9,34,35^:   - Hb <11g/dl irrespective of sex - Hb ≥2g/dl decrease from baseline if already anaemic at presentation. - Anaemia of any degree with requirement for red cell transfusion as determined by clinical team.   If participant was anaemic at baseline, then they were considered to have developed anaemia only if they fulfilled either of the latter 2 criteria. |
| Thrombocytopenia | Platelet count < 100 x 10^9^/l at any point in the study.^36^ The lifespan of platelets ranges from 7 to 10 days.^37^ Participants were therefore considered to have thrombocytopenia at each weekly scheduled visit once the platelet count was below the threshold and represented a decrease from baseline or a decrease at any point during follow-up after a rise to normal.  Participants with thrombocytopenia at baseline were therefore considered to have thrombocytopenia if the subsequent weekly platelet counts were lower than baseline counts. |
| Tumour Lysis Syndrome | Defined according to the Cairo and Bishop Classification system.^38^ |
| Acute pancreatitis | Defined according to the Atlanta criteria: presence of at least two of the following three criteria: clinical presentation resembling pancreatitis, amylase or lipase more than three times the upper normal level and imaging compatible with acute pancreatitis.^39^ |
| Febrile neutropenia | Axillary temperature measuring >38^o^C on one occasion or >37.5^o^C for at least one hour in patients with an absolute neutrophil count (ANC) of <0.5x10^9^/L, or who had an ANC of between 0.5 and 1x10^9^/L, and whose ANC was expected to decrease below 0.5x10^9^/L within 24–48 hours (POU KBTH Supportive care guidelines 2021). |
| Sepsis | “Systemic inflammatory response syndrome (SIRS) in the presence of suspected or culture- proven infection”. SIRS was diagnosed in the presence of two or more of the following (one of which must be abnormal temperature or WBC)^40^:   - Axillary temperature of >37.5°C or <36°C - Tachycardia (heart rate above the following thresholds for the stated age groups: 140 beats/minute (>1 to 5 years), 130 beats/minute (>5 to 12 years), 110 beats/minute (>12 to 17years) - Respiratory rate above the following thresholds for the stated age groups: 22 breaths/minute (>1 to 5 years), 18 breaths/minute (>5 to 12 years), 14 breaths/minute (>12 to 17years) - WBC count increased or decreased for age. |
| Mucositis | Diagnosed on clinical examination of the oral mucosa for erythema and/or ulcers.^41^ To grade mucositis according to the NCI CTCAE v5.0 classification, participants were also asked about associated symptoms: pain, interference with oral intake and/or need for modification of diet.^9^ |
| Gastritis | Presence of abdominal pain (particularly epigastric) and/or epigastric tenderness on examination, with or without the following associated symptoms: nausea, vomiting, loss of appetite or bloating.^42^ |
| Stroke | Defined as a focal (or global) neurologic impairment of sudden onset and lasting more than 24 hours (or leading to death), and of presumed vascular origin. Stroke was diagnosed clinically in the presence of any of the following definite focal or global disturbances of cerebral function: unilateral or bilateral motor impairment, aphasia/dysphasia, ataxia of acute onset.^43^ |
